# Synthesis, crystal structure and thermal properties of di-μ-iodido-bis­[bis­(2-chloro­pyrazine-κ*N*)copper(I)]

**DOI:** 10.1107/S2056989023001238

**Published:** 2023-02-17

**Authors:** Christian Näther, Inke Jess

**Affiliations:** a Institut für Anorganische Chemie, Universität Kiel, Max-Eyth.-Str. 2, 24118 Kiel, Germany; Universidad Nacional Autónoma de México, México

**Keywords:** synthesis, crystal structure, binuclear complex, thermal properties

## Abstract

Reaction of copper(I) iodide in pure 2-chloro­pyrazine leads to the formation of a few crystals of the title compound, [Cu_2_I_2_(C_4_H_3_ClN_2_)_4_] or (CuI)_2_(2-chloro­pyrazine)_4_, which was characterized by single-crystal X-ray diffraction. In its crystal structure, the Cu^I^ cations are each tetra­hedrally coordinated by two iodide anions and two 2-chloro­pyrazine ligands and are linked into binuclear complexes consisting of (CuI)_2_ rings located on centers of inversion. PXRD investigations of a few crystals obtained from the suspension indicate that the title compound is contaminated with a small amount of the 2-chloro­pyrazine-deficient compound CuI(2-chloro­pyrazine) already reported in the literature. PXRD investigations prove that the title compound immediately decomposes at room temperature into CuI(2-chloro­pyrazine) and this might be the reason why no pure samples can be obtained. TDA–TG–MS investigations shows two mass losses, the first of which corresponds to the formation of CuI(2-chloro­pyrazine), whereas in the second mass loss CuI is formed.

## Abstract 2

In the crystal structure of the title compound, the copper(I) cations are each tetra­hedrally coordinated by two 2-chloro­pyrazine ligands and two iodide anions and linked into binuclear complexes by pairs of μ-1,1-bridging iodide anions.

## 1. Chemical context

Coordination compounds based on transition-metal halides show a versatile structural behavior, which is observed particularly in compounds that contain Cu^I^ cations (Kromp & Sheldrick, 1999 ▸; Peng *et al.*, 2010 ▸; Li *et al.*, 2005 ▸; Näther & Jess, 2004 ▸). These compounds are also of inter­est because of their luminescence behavior (Gibbons *et al.*, 2017 ▸; Mensah *et al.*, 2022 ▸). For one given metal halide Cu*X* (*X* = Cl, Br, I) and one specific neutral coligand, several compounds are usually observed that differ in the ratio between the metal halide and the coligand – this is the reason why so many compounds with different Cu*X* (*X* = Cl, Br, I) substructures (such as, for example, dimers, single and double chains or layers) are observed that can be further connected into more condensed networks if bridging neutral coligands are used in the synthesis. In general, it is observed that with decreasing amounts of the coligand, the synthesis leads to the formation of compounds with more condensed Cu*X* substructures. In this context, it is noted that upon heating, the most coligand-rich compounds usually lose their coligands stepwise and transform into coligand-deficient phases and that this is not limited to Cu^I^, but can also be expanded to Cd^II^ and Zn^II^ compounds (Näther *et al.*, 2001 ▸, 2007 ▸, 2017 ▸; Näther & Jess, 2001 ▸). This can easily be investigated by thermogravimetry of the most coligand-rich compounds, where each mass loss corresponds to the formation of a new coligand-deficient phase with a more condensed Cu*X* substructure. Surprisingly, even for compounds with the same Cu*X*:ligand ratio, sometimes a different thermal reactivity is observed. This is the case, for example, for compounds based on Cu*X* (*X* = Cl, Br, I) and 2-chloro­pyrazine as ligands with the general composition Cu*X*(2-chloro­pyrazine) (*X* = Cl, Br, I; Näther, Wriedt & Jess, 2002 ▸; Näther, Greve & Jess, 2002 ▸). In the isotypic chloride and bromide compounds, the copper cations are tetra­hedrally coordinated by two bridging 2-chloro­pyrazine ligands and two halide anions. The cations are linked by single μ-1,1-bridging halide anions into chains that are further connected into layers by μ-1,4-bridging 2-chloro­pyrazine ligands (Fig. S1 in the supporting information). In contrast, in CuI(2-chloro­pyrazine), each copper cation is tetra­hedrally coordinated by three iodide anions and only one terminal N-bonding 2-chloro­pyrazine ligand that is coordinated to the copper center by the N atom that is not adjacent to the chloro substituent. The cations are linked into double chains *via* bridging iodide anions (Fig. S1). If the chloride and the bromide compounds are heated, all 2-chloro­pyrazine ligands are removed in a single step, leading to the formation of Cu*X* (*X* = Cl, Br). In contrast, the iodide compound decomposes in two discrete steps, where in the first step only half of the coligands are removed, leading to the formation of (CuI)_2_(2-chloro­pyrazine), which decomposes into CuI upon further heating (Näther, Greve & Jess, 2002 ▸). 
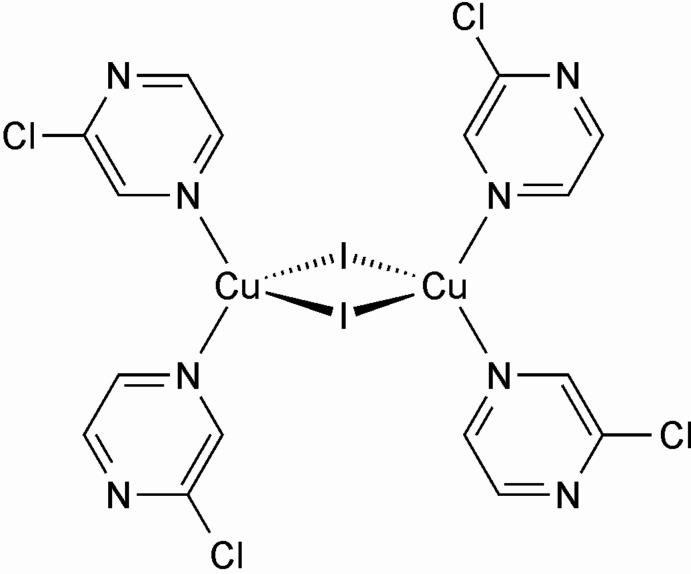


Concerning the composition of all of these compounds, in principle, more 2-chloro­pyrazine-rich compounds with the composition Cu*X*(2-chloro­pyrazine)_2_ might exist, in which, according to simple chemical considerations, each two copper cations would be tetra­hedrally coordinated by two halide anions and two N-terminal 2-chloro­pyrazine ligands and linked into binuclear complexes by pairs of μ-1,1-bridging halide anions. One might argue that this arrangement is less stable compared to that with bridging 2-chloro­pyrazine ligands, but one should keep in mind that both N atoms of this ligand are not equivalent, because coordination to the N atom that is adjacent to the chloro substituent is sterically hindered. That this coordination exists is obvious from the crystal structure of (CuI)(2-chloro­pyrazine) mentioned above, even if this Cu*X* substructure is different. Moreover, a few compounds with such a structure have already been reported in the literature, including, for example, (CuI)_2_(2-cyano­pyrazine)_4_ (Refcodes: DINQIA and DINQIA01; Rossenbeck & Sheldrick, 1999 ▸ and Jana *et al.*, 2016 ▸), (CuI)_2_(2-ethyl­pyrazine)_4_ (Refcode: EMELEN; Näther *et al.*, 2003 ▸), (CuI)_2_-(methyl­sulfanyl­pyrazine)_4_ (Refcode: QOWYOT; Artem’ev *et al.*, 2019 ▸) and (CuI)_2_(2,2′-biquinoxaline) (Refcode: RIXGEL; Fitchett & Steel, 2008 ▸), all with iodide as counter-anion.

To check if such a compound can be synthesized, all three copper(I) halides were reacted in different solvents with a very large excess of 2-chloro­pyrazine, but no new crystalline phases were observed. On the contrary, if CuI is reacted as a suspension in pure 2-chloro­pyrazine, yellow-colored crystals of a new crystalline phase are obtained. In contrast, with CuCl or CuBr only the known compounds Cu*X*(2-chloro­pyrazine) with *X* = Cl, Br are obtained. Single-crystal structure analysis proved that a new compound with the composition (CuI)_2_(2-chloro­pyrazine)_4_ has been obtained.

## 2. Structural commentary

**Figure 1:**
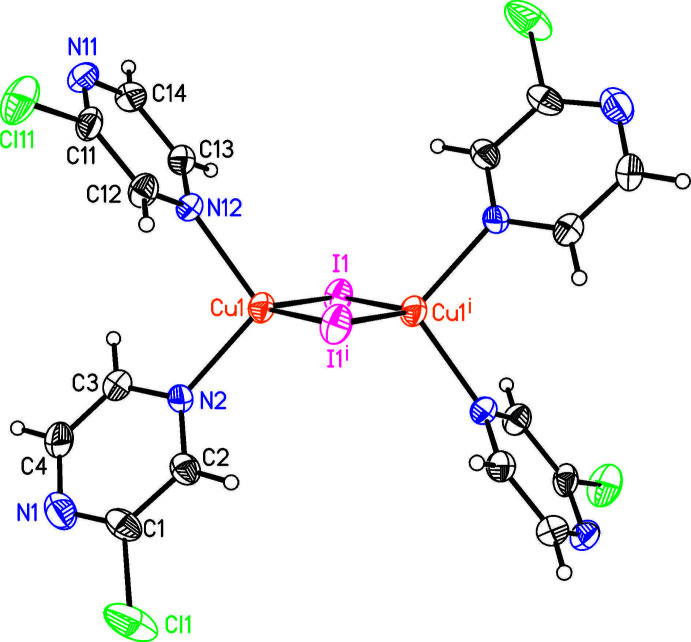
Crystal structure of the title compound with atom labeling and displacement ellipsoids drawn at the 50% probability level. Symmetry codes: (i) −*x* + 2, −*y* + 1, −*z* + 2.

The asymmetric unit of the title compound (CuI)_2_(2-chloro­pyrazine)_4_ consists of one copper(I) cation, one iodide anion and two 2-chloro­pyrazine ligands that are located in general positions. The copper(I) cations are tetra­hedrally coordinated by two symmetry-related iodide anions and two crystallographically independent 2-chloro­pyrazine ligands (Fig. 1 ▸). Each two copper(I) cations are linked by pairs of μ-1,1-bridging iodide anions into binuclear complexes consisting of four-membered (CuI)_2_ rings located on centers of inversion. The Cu—Cu distance within these rings amounts to 2.5643 (10) Å (Table 1 ▸). Bond lengths and angles are similar to those in related compounds and show that the tetra­hedra are strongly distorted (Table 1 ▸).

This structure is similar to those of (CuI)_2_(2-cyano­pyrazine)_4_ (Rossenbeck & Sheldrick, 1999 ▸; Jana *et al.*, 2016 ▸), (CuI)_2_(2-ethyl­pyrazine)_4_ (Näther *et al.*, 2003 ▸), (CuI)_2_-(methyl­sulfanyl­pyrazine)_4_ (Artem’ev *et al.*, 2019 ▸) and (CuI)_2_(2,2′-biquinoxaline) (Fitchett & Steel, 2008 ▸) already reported in the literature, which also form binuclear complexes with (CuI)_2_ rings as the main structural motif.

## 3. Supra­molecular features

**Figure 2:**
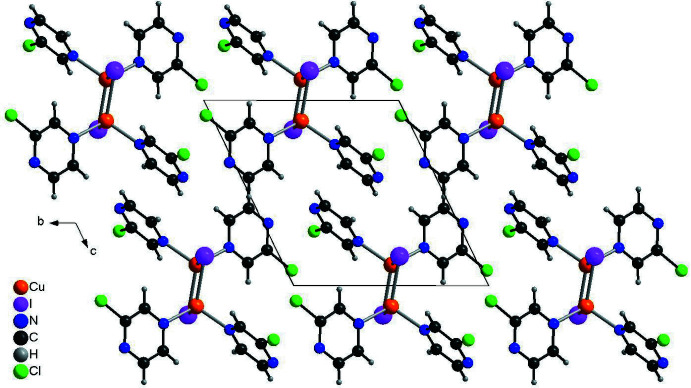
Crystal structure of the title compound in a view along the crystallographic *a*-axis.

In the crystal structure of the title compound, the binuclear complexes are arranged in columns that propagate along the crystallographic *a*-axis direction (Fig. 2 ▸). No directional inter­molecular inter­actions occur between the complexes. One C—H⋯N and one C—H⋯I contact is observed, but their distances and angles indicate that they do not correspond to significant inter­actions (Table 2 ▸).

## 4. Powder X-ray diffraction and thermoanalytical investigations

**Figure 3:**
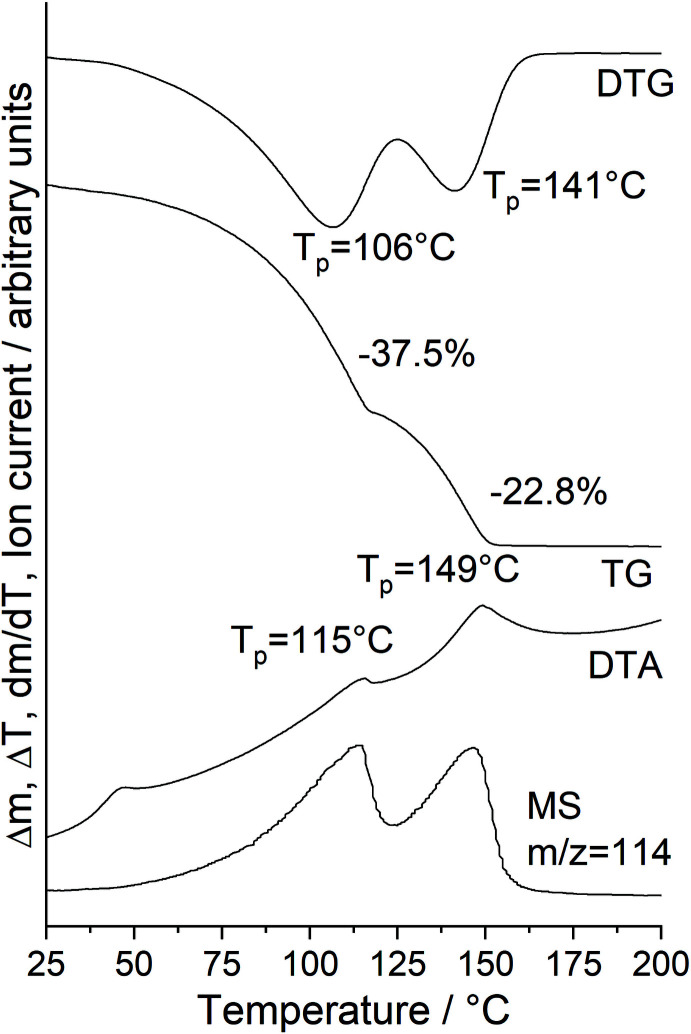
DTG, TG, DTA and MS trend scan curves for the title compound measured with a heating rate of 4°C min^−1^.

**Figure 4:**
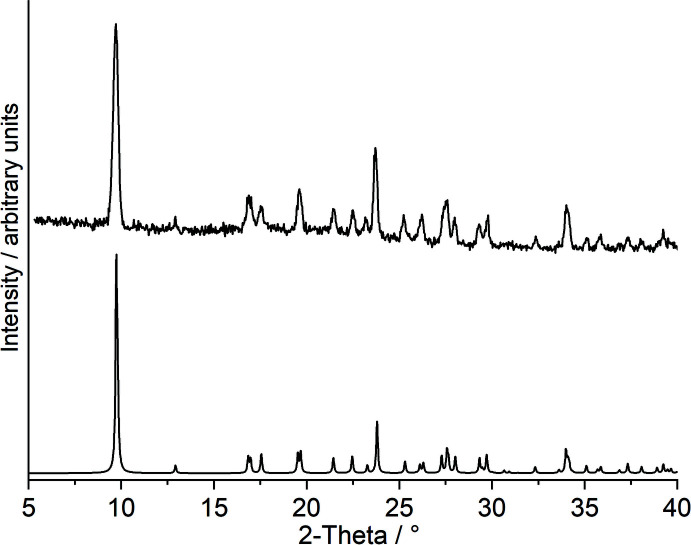
Experimental PXRD pattern of the residue obtained after the first mass loss in a DTA–TG–MS measurement of the title compound (top) and PXRD pattern calculated for CuI(2-chloro­pyrazine) (bottom).

Further investigations prove that the unreacted 2-chloro­pyrazine cannot be removed by filtration and washing because immediate decomposition is observed. Nevertheless, XRPD investigations reveal that most of the sample consists of crystals of the title compound, even if all of the powder patterns are of very low quality, which can be traced back to the instability of this compound and to the fact that only very small amounts of crystals were obtained and these were embedded in pure 2-chloro­pyrazine and that grinding of such samples leads to the formation of an amorphous phase (Fig. S2). Careful inspection of the powder pattern indicates that this sample is contaminated at least with CuI(2-chloro­pyrazine) reported in the literature (Näther, Greve & Jess, 2002 ▸). This indicates that the title compound has already decomposed into this compound at room temperature. To prove this assumption, freshly prepared crystals were stored at room temperature overnight and were afterwards investigated by PXRD, confirming that the title compound has been completely transformed into the ligand-deficient compound CuI(2-chloro­pyrazine) (Fig. S3). These observations indicate that CuI(2-chloro­pyrazine) with a bridging coordination of the 2-chloro­pyrazine ligand is more stable than the title compound, in which the 2-chloro­pyrazine acts as a terminal ligand. Additional DTA–TG–MS investigations reveal that the title compound loses two 2-chloro­pyrazine ligands in two subsequent steps, in which 2-chloro­pyrazine is always removed (*m*/*z* = 114, Fig. 3 ▸). The experimental mass loss in the first step (Δ*m*_exp ._ = 37.5%) is much larger than that expected for the removal of all of the 2-chloro­pyrazine ligands from the title compound (Δ*m*_calc._ = 19.1%), which originates from the fact that the 2-chloro­pyrazine coating the crystals cannot be removed. However, PXRD of the residue obtained after the first mass loss confirms that CuI(2-chloro­pyrazine) is formed as an inter­mediate (Fig. 4 ▸). It is noted that no additional step is observed that would correspond to the formation of the most 2-chloro­pyrazine-deficient compound, (CuI)_2_(2-chloro­pyrazine), because this event would happen at a much lower temperature, whereas our measurements indicate an excess of 2-chloro­pyrazine is still present in the gas phase. Finally, the product formed after the second mass loss was also investigated py PXRD, which proves that CuI (Hull, & Keen, 1994 ▸) is formed in this step (Fig. S4).

## 5. Database survey

A search in the CCDC database (Groom *et al.*, 2016 ▸, CSD Version 5.43, March 2022) for compounds with a structure similar to that of the title compound revealed several hits, including (CuI)_2_(2-cyano­pyrazine)_4_ (Refcodes: DINQIA and DINQIA01; Rossenbeck & Sheldrick, 1999 ▸; Jana *et al.*, 2016 ▸), (CuI)_2_(2-ethyl­pyrazine)_4_ (Refcode: EMELEN; Näther *et al.*, 2003 ▸), (CuI)_2_-(methyl­sulfanyl­pyrazine)_4_ (Refcode: QOWYOT; Artem’ev *et al.*, 2019 ▸) and (CuI)_2_(2,2′-biquinoxaline) (Refcode: RIXGEL; Fitchett & Steel, 2008 ▸).

A further search for compounds based on copper halides and 2-chloro­pyrazine as ligand lead to only a very few compounds. They include the three compounds with the composition Cu*X*(2-chloro­pyrazine) with *X* = Cl, Br, I mentioned above (Refcodes: ODOFES, ODOFIW and ODOFOC; Näther, Wriedt & Jess 2002 ▸) as well as two isotypic discrete complexes with the composition Cu*X*_2_(2-chloro­pyrazine)_2_ with *X* = Cl, Br that contain Cu^II^ cations (Refcodes: FULYIV and FULYOB; Herringer *et al.*, 2010 ▸).

Some related compounds can also be found with 2-meth­yl­pyrazine as coligand because the exchange of a chloro atom by a methyl group sometimes leads to compounds with similar crystal structures as the van der Waals radius of a chlorine atom is comparable to that of a methyl group (Desiraju & Sarma, 1986 ▸). This is obvious from Cu*X*(2-methyl­pyrazine) with *X* = Cl, Br (Refcodes: XEBMOG and XEBMIA; Rossenbeck & Sheldrick, 2000 ▸), in which the copper(I) cations are linked by μ-1,1-bridging halide anions into chains that are further connected into layers by bridging 2-methyl­pyrazine ligands. This structure is identical to that of Cu*X*(2-chloro­pyrazine) (*X* = Cl, Br). Moreover, both the 2-methyl­pyrazine and the 2-chloro­pyrazine compounds crystallize in the monoclinic space group *P*2_1_/*c* with very similar lengths of the unit-cell axes, but with a significantly different β angle. In this context it is noted that with 2-methyl­pyrazine, two coligand-deficient compounds with the composition (Cu*X*)_2_(2-methyl­pyrazine) with *X* = Br, I (Refcodes: XEBMUM and XEBNAT; Rossenbeck & Sheldrick, 2000 ▸) were observed that could not be prepared with 2-chloro­pyrazine.

Furthermore, the isotypic compounds (Cu*X*)_2_(2-methyl­pyrazine)(tri­phenyl­phosphine)_2_ aceto­nitrile solvate with *X* = Br, I [Refcodes: AKOPOI (Kuwahara *et al.*, 2020 ▸) and RAYXAT (Liu *et al.*, 2017 ▸)], in which each copper cation is tetra­hedrally coordinated by one 2-methyl­pyrazine and one tri­methyl­phoshine ligand as well as two halide anions, are known. Similarly to the title compound, both copper cations are linked by two bridging halide anions into (CuI)_2_ rings, but strikingly the binuclear units are linked by the 2-methyl­pyrazine ligands into chains. Moreover, (CuI)_2_(2-methyl­pyrazine)_2_-2-methyl­pyrazine solvate (XEBMEW; Rossenbeck & Sheldrick, 2000 ▸) also contains (CuI)_2_ rings.

Finally, two compounds with the composition Cu*X*(2-methyl­pyrazine)(tri­phenyl­phosphine)_2_ with *X* = Cl, I [Refcodes: KAMKER (Ohara, *et al.*, 2017 ▸) and NAKYIL (Kondo *et al.*, 2020 ▸)] are reported, which form discrete complexes. This structural motif is unknown for compounds based on Cu*X* and 2-chloro­pyrazine.

## 6. Synthesis and crystallization

**Synthesis**

CuI and 2-chloro­pyrazine were purchased from Sigma-Aldrich and used as received.

Yellow-colored single crystals suitable for single-crystal X-ray analysis were obtained within three days by the reaction of 0.5 mmol (95.23 mg) of CuI and 2 mL of 2-chloro­pyrazine. No stoichiometric ratios can be used as an excess of 2-chloro­pyrazine is needed because it acts as reactant and solvent. The additional 2-chloro­pyrazine cannot be removed by filtration and washing, because this leads immediately to the transformation of the title compound into CuI(2-chloro­pyrazine).

**Experimental details**

The data collection for single crystal structure analysis was performed using an Imaging Plate Diffraction System (IPDS-1) from Stoe with Mo *K*α radiation.

The PXRD measurements were performed with Cu *K*α_1_ radiation (λ = 1.540598 Å) using a Stoe Transmission Powder Diffraction System (STADI P) equipped with a MYTHEN 1K detector and a Johansson-type Ge(111) monochromator.

Differential thermoanalysis and thermogravimetry coupled to mass spectrometry (DTA–TG–MS) investigations were performed with a STA-429 thermobalance from Netzsch with skimmer coupling to a quadrupole mass spectrometer from Balzers. The measurements were performed in a dynamic nitro­gen atmosphere in Al_2_O_3_ crucibles with a heating rate of 4°C min^−1^. The instrument was calibrated using standard reference materials.

## 7. Refinement

Crystal data, data collection and structure refinement details are summarized in Table 3 ▸. The C—H hydrogen atoms were positioned in an idealized geometry and refined isotropically with *U_i_*_so_(H) = 1.2*U*_eq_(C).

## Supplementary Material

Crystal structure: contains datablock(s) I. DOI: 10.1107/S2056989023001238/jq2025sup1.cif

Structure factors: contains datablock(s) I. DOI: 10.1107/S2056989023001238/jq2025Isup2.hkl

Fig. S1. Crystal structure of CuX(2-chloropyrazine) (X = Cl, Br) with the CuBr compound as representative (left) and of CuI(2-chloropyrazine) (right) drawn based on the data given in reference Nather et al., 2002. DOI: 10.1107/S2056989023001238/jq2025sup3.jpg

Fig. S2. Experimental PXRD pattern of freshly prepared crystals of the title compound (top), together with the calculated powder pattern for the title compound (mid) and CuI(2-chloropyrazine) (bottom) retrieved from literature (Nather et al., 2002). DOI: 10.1107/S2056989023001238/jq2025sup4.jpg

Fig. S3. Experimental PXRD pattern of the residue obtained by storing freshly prepared crystals of the title compound for 1 d at room-temperature (top) and calculated powder pattern for CuI(2-chloropyrazine) (bottom) retrieved from literature (Nather et al., 2002). DOI: 10.1107/S2056989023001238/jq2025sup5.jpg

Fig. S4. Experimental PXRD pattern of the residue obtained at 200C in a TG-DTA-MS measurement of the title compound (top) and calculated pattern for CuI retrieved from literature (Hull and Keen, 1994). DOI: 10.1107/S2056989023001238/jq2025sup6.jpg

CCDC reference: 2241117

Additional supporting information:  crystallographic information; 3D view; checkCIF report

## Figures and Tables

**Table 1:** Selected geometric parameters (Å, °)

Cu1—N2	2.070 (3)	Cu1—I1	2.6093 (5)
Cu1—N12	2.078 (3)	Cu1—I1^i^	2.6476 (6)
Cu1—Cu1^i^	2.5643 (10)		
			
N2—Cu1—N12	103.01 (12)	N2—Cu1—I1^i^	107.58 (9)
N2—Cu1—I1	109.42 (9)	N12—Cu1—I1^i^	105.80 (9)
N12—Cu1—I1	107.83 (9)	I1—Cu1—I1^i^	121.61 (2)

Symmetry code: (i) 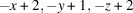 .

**Table 2:** Hydrogen-bond geometry (Å, °)

*D*—H⋯*A*	*D*—H	H⋯*A*	*D*⋯*A*	*D*—H⋯*A*
C3—H3⋯N11^ii^	0.94	2.68	3.241 (5)	119
C13—H13⋯I1	0.94	3.24	3.872 (4)	127

Symmetry code: (ii) 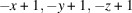 .

**Table 3:** Experimental details

Crystal data	
Chemical formula	[Cu_2_I_2_(C_4_H_3_ClN_2_)_4_]
*M*_r_	839.02
Crystal system, space group	Triclinic, *P* 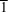
Temperature (K)	220
*a*, *b*, *c* (Å)	7.5220 (6), 10.1067 (8), 10.1973 (9)
α, β, γ (°)	108.932 (9), 101.922 (10), 111.088 (9)
*V* (Å^3^)	636.62 (11)
*Z*	1
Radiation type	Mo *K*α
μ (mm^−1^)	4.54
Crystal size (mm)	0.25 × 0.20 × 0.18

Data collection	
Diffractometer	Stoe *IPDS1*
Absorption correction	Numerical (*X-SHAPE* and *X-RED* 32; Stoe, 2008 ▸)
*T*_min_, *T*_max_	0.556, 0.667
No. of measured, independent and observed [*I* > 2σ(*I*)] reflections	6865, 3018, 2670
*R*_int_	0.041

Refinement	
*R*[*F* ^2^ > 2σ(*F* ^2^)], *wR*(*F* ^2^), *S*	0.037, 0.100, 1.02
No. of reflections	3018
No. of parameters	146
H-atom treatment	H-atom parameters constrained
Δρ_max_, Δρ_min_ (e Å^−3^)	1.22, −1.91

Computer programs: *X-AREA* (Stoe, 2008 ▸), *SHELXT2014/5* (Sheldrick, 2015*a* ▸), *SHELXL2016/6* (Sheldrick, 2015*b* ▸), *DIAMOND* (Brandenburg, 1999 ▸) and *publCIF* (Westrip, 2010 ▸).

## supplementary crystallographic information

### Crystal data

**Table:**

[Cu_2_I_2_(C_4_H_3_ClN_2_)_4_]	*Z* = 1
*M**_r_* = 839.02	*F*(000) = 396
Triclinic, *P*1	*D*_x_ = 2.188 Mg m^−^^3^
*a* = 7.5220 (6) Å	Mo *K*α radiation, λ = 0.71073 Å
*b* = 10.1067 (8) Å	Cell parameters from 6865 reflections
*c* = 10.1973 (9) Å	θ = 2.5–28.1°
α = 108.932 (9)°	µ = 4.54 mm^−^^1^
β = 101.922 (10)°	*T* = 220 K
γ = 111.088 (9)°	Block, yellow
*V* = 636.62 (11) Å^3^	0.25 × 0.20 × 0.18 mm

### Data collection

**Table:**

Stoe IPDS-1 diffractometer	2670 reflections with *I* > 2σ(*I*)
Phi scans	*R*_int_ = 0.041
Absorption correction: numerical (X-Shape and X-Red 32; Stoe, 2008)	θ_max_ = 28.1°, θ_min_ = 2.5°
*T*_min_ = 0.556, *T*_max_ = 0.667	*h* = −9→9
6865 measured reflections	*k* = −13→13
3018 independent reflections	*l* = −13→13

### Refinement

**Table:**

Refinement on *F*^2^	Hydrogen site location: inferred from neighbouring sites
Least-squares matrix: full	H-atom parameters constrained
*R*[*F*^2^ > 2σ(*F*^2^)] = 0.037	*w* = 1/[σ^2^(*F*_o_^2^) + (0.0731*P*)^2^] where *P* = (*F*_o_^2^ + 2*F*_c_^2^)/3
*wR*(*F*^2^) = 0.100	(Δ/σ)_max_ = 0.001
*S* = 1.01	Δρ_max_ = 1.22 e Å^−^^3^
3018 reflections	Δρ_min_ = −1.91 e Å^−^^3^
146 parameters	Extinction correction: *SHELXL2016/6* (Sheldrick 2015b), Fc^*^=kFc[1+0.001xFc^2^λ^3^/sin(2θ)]^-1/4^
0 restraints	Extinction coefficient: 0.043 (3)

### Special details

**Table:**

Geometry. All esds (except the esd in the dihedral angle between two l.s. planes) are estimated using the full covariance matrix. The cell esds are taken into account individually in the estimation of esds in distances, angles and torsion angles; correlations between esds in cell parameters are only used when they are defined by crystal symmetry. An approximate (isotropic) treatment of cell esds is used for estimating esds involving l.s. planes.

### Fractional atomic coordinates and isotropic or equivalent isotropic displacement parameters (Å^2^)

**Table:**

	*x*	*y*	*z*	*U*_iso_*/*U*_eq_	
Cu1	0.83699 (6)	0.44607 (6)	0.88784 (5)	0.02577 (15)	
I1	1.13274 (3)	0.38430 (3)	0.84123 (3)	0.02918 (13)	
N1	0.1826 (5)	−0.0235 (4)	0.6635 (5)	0.0382 (8)	
C1	0.2875 (6)	0.0483 (5)	0.8075 (5)	0.0310 (8)	
C2	0.4784 (6)	0.1784 (5)	0.8761 (5)	0.0273 (7)	
H2	0.545096	0.224589	0.980055	0.033*	
N2	0.5673 (4)	0.2377 (4)	0.7947 (4)	0.0240 (6)	
C3	0.4652 (6)	0.1671 (5)	0.6476 (5)	0.0352 (9)	
H3	0.524713	0.206273	0.587078	0.042*	
C4	0.2745 (7)	0.0380 (6)	0.5828 (5)	0.0400 (10)	
H4	0.206856	−0.008357	0.478949	0.048*	
Cl1	0.1760 (2)	−0.02696 (16)	0.91711 (16)	0.0573 (4)	
N11	0.6729 (5)	0.7005 (4)	0.5844 (4)	0.0312 (7)	
C11	0.5985 (6)	0.6885 (4)	0.6870 (4)	0.0271 (8)	
C12	0.6478 (6)	0.6211 (5)	0.7776 (4)	0.0270 (7)	
H12	0.589696	0.617235	0.850384	0.032*	
N12	0.7785 (4)	0.5614 (4)	0.7617 (3)	0.0238 (6)	
C13	0.8562 (5)	0.5723 (5)	0.6574 (4)	0.0267 (7)	
H13	0.948559	0.531426	0.643161	0.032*	
C14	0.8049 (6)	0.6421 (5)	0.5694 (5)	0.0311 (8)	
H14	0.864111	0.648491	0.497602	0.037*	
Cl11	0.42309 (19)	0.76067 (15)	0.70711 (14)	0.0455 (3)	

### Atomic displacement parameters (Å^2^)

**Table:**

	*U*^11^	*U*^22^	*U*^33^	*U*^12^	*U*^13^	*U*^23^
Cu1	0.0206 (2)	0.0312 (3)	0.0281 (3)	0.01328 (19)	0.00699 (17)	0.0157 (2)
I1	0.02846 (17)	0.03876 (19)	0.02799 (18)	0.02343 (12)	0.01185 (11)	0.01347 (12)
N1	0.0280 (16)	0.0314 (19)	0.043 (2)	0.0099 (14)	0.0061 (15)	0.0109 (16)
C1	0.0267 (17)	0.0208 (17)	0.043 (2)	0.0098 (14)	0.0161 (16)	0.0099 (16)
C2	0.0249 (17)	0.0229 (17)	0.0288 (19)	0.0099 (14)	0.0075 (14)	0.0080 (15)
N2	0.0185 (13)	0.0233 (15)	0.0279 (16)	0.0083 (11)	0.0045 (11)	0.0124 (13)
C3	0.0318 (19)	0.039 (2)	0.032 (2)	0.0127 (17)	0.0053 (16)	0.0214 (19)
C4	0.035 (2)	0.038 (2)	0.030 (2)	0.0087 (18)	−0.0033 (16)	0.0141 (19)
Cl1	0.0531 (7)	0.0426 (7)	0.0509 (7)	−0.0019 (5)	0.0312 (6)	0.0111 (6)
N11	0.0345 (17)	0.0280 (17)	0.0299 (17)	0.0132 (14)	0.0054 (13)	0.0166 (14)
C11	0.0267 (17)	0.0237 (17)	0.0264 (19)	0.0141 (14)	0.0013 (14)	0.0082 (15)
C12	0.0266 (17)	0.035 (2)	0.0263 (18)	0.0188 (15)	0.0113 (14)	0.0151 (16)
N12	0.0230 (14)	0.0247 (15)	0.0257 (15)	0.0123 (12)	0.0065 (11)	0.0135 (13)
C13	0.0230 (16)	0.0296 (19)	0.0288 (19)	0.0128 (14)	0.0079 (14)	0.0143 (16)
C14	0.0314 (18)	0.031 (2)	0.030 (2)	0.0118 (16)	0.0097 (15)	0.0162 (17)
Cl11	0.0526 (6)	0.0525 (7)	0.0470 (6)	0.0421 (6)	0.0143 (5)	0.0220 (5)

### Geometric parameters (Å, º)

**Table:**

Cu1—N2	2.070 (3)	C3—H3	0.9400
Cu1—N12	2.078 (3)	C4—H4	0.9400
Cu1—Cu1^i^	2.5643 (10)	N11—C11	1.302 (6)
Cu1—I1	2.6093 (5)	N11—C14	1.336 (6)
Cu1—I1^i^	2.6476 (6)	C11—C12	1.380 (5)
N1—C1	1.313 (6)	C11—Cl11	1.739 (4)
N1—C4	1.342 (6)	C12—N12	1.336 (5)
C1—C2	1.382 (5)	C12—H12	0.9400
C1—Cl1	1.735 (4)	N12—C13	1.330 (5)
C2—N2	1.327 (5)	C13—C14	1.384 (6)
C2—H2	0.9400	C13—H13	0.9400
N2—C3	1.335 (5)	C14—H14	0.9400
C3—C4	1.378 (6)		
			
N2—Cu1—N12	103.01 (12)	N2—C3—H3	119.3
N2—Cu1—Cu1^i^	130.55 (9)	C4—C3—H3	119.3
N12—Cu1—Cu1^i^	126.35 (9)	N1—C4—C3	121.9 (4)
N2—Cu1—I1	109.42 (9)	N1—C4—H4	119.0
N12—Cu1—I1	107.83 (9)	C3—C4—H4	119.0
Cu1^i^—Cu1—I1	61.554 (19)	C11—N11—C14	115.8 (3)
N2—Cu1—I1^i^	107.58 (9)	N11—C11—C12	124.4 (4)
N12—Cu1—I1^i^	105.80 (9)	N11—C11—Cl11	116.8 (3)
Cu1^i^—Cu1—I1^i^	60.06 (2)	C12—C11—Cl11	118.7 (3)
I1—Cu1—I1^i^	121.61 (2)	N12—C12—C11	119.8 (3)
Cu1—I1—Cu1^i^	58.39 (2)	N12—C12—H12	120.1
C1—N1—C4	115.1 (4)	C11—C12—H12	120.1
N1—C1—C2	124.4 (4)	C13—N12—C12	116.9 (3)
N1—C1—Cl1	117.0 (3)	C13—N12—Cu1	123.3 (3)
C2—C1—Cl1	118.6 (3)	C12—N12—Cu1	119.7 (3)
N2—C2—C1	119.7 (4)	N12—C13—C14	121.8 (4)
N2—C2—H2	120.1	N12—C13—H13	119.1
C1—C2—H2	120.1	C14—C13—H13	119.1
C2—N2—C3	117.4 (3)	N11—C14—C13	121.3 (4)
C2—N2—Cu1	122.7 (3)	N11—C14—H14	119.3
C3—N2—Cu1	119.5 (3)	C13—C14—H14	119.3
N2—C3—C4	121.4 (4)		

Symmetry code: (i) −*x*+2, −*y*+1, −*z*+2.

### Hydrogen-bond geometry (Å, º)

**Table:**

*D*—H···*A*	*D*—H	H···*A*	*D*···*A*	*D*—H···*A*
C3—H3···N11^ii^	0.94	2.68	3.241 (5)	119
C13—H13···I1	0.94	3.24	3.872 (4)	127

Symmetry code: (ii) −*x*+1, −*y*+1, −*z*+1.
