# Supplementary figures and images for: Synthesis, crystal structure and thermal properties of di-μ-iodido-bis­[bis­(2-chloro­pyrazine-κN)copper(I)]
Source: Acta Crystallogr E Crystallogr Commun. 2023 Feb 17;79(Pt 3):167–71. doi: 10.1107/S2056989023001238 (PMC9993928; doi:10.1107/S2056989023001238)

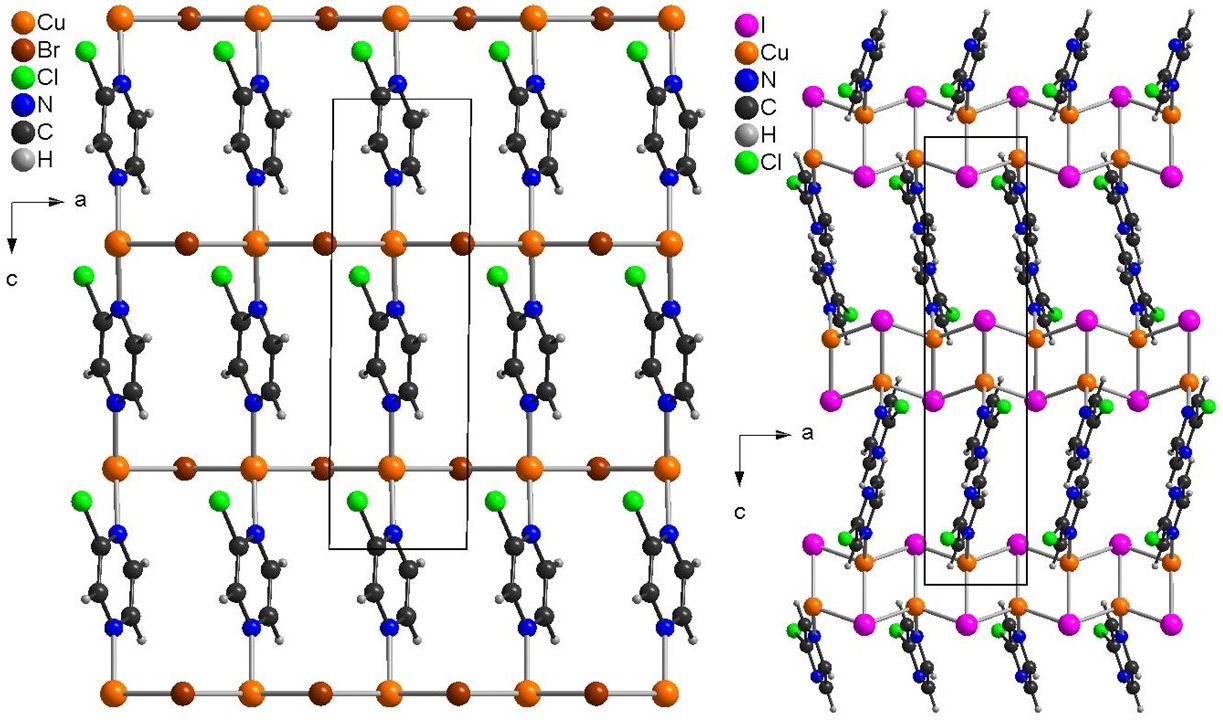

Supplement: Supplementary file 3 [file e-79-00167-sup3.jpg]

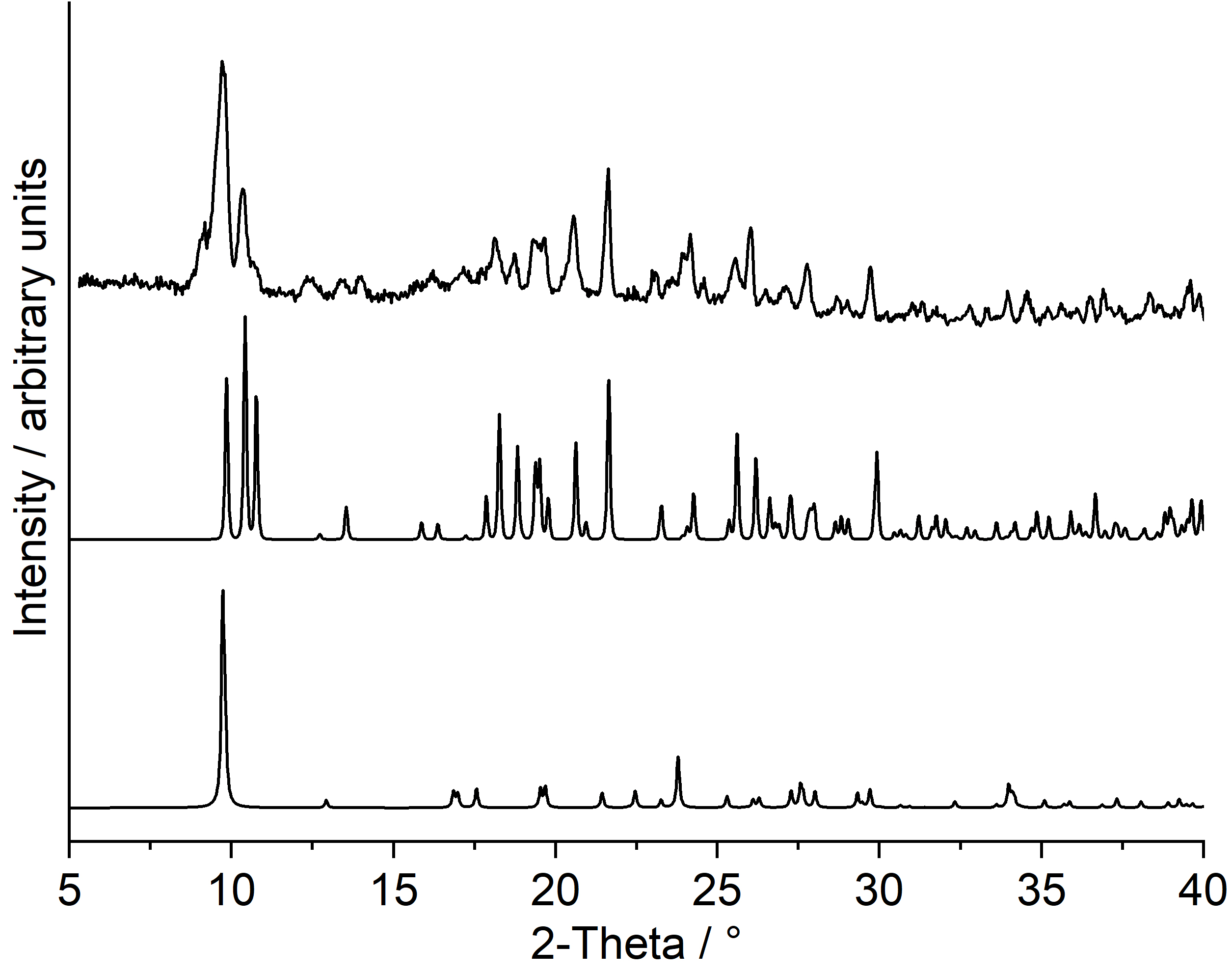

Supplement: Supplementary file 4 [file e-79-00167-sup4.jpg]

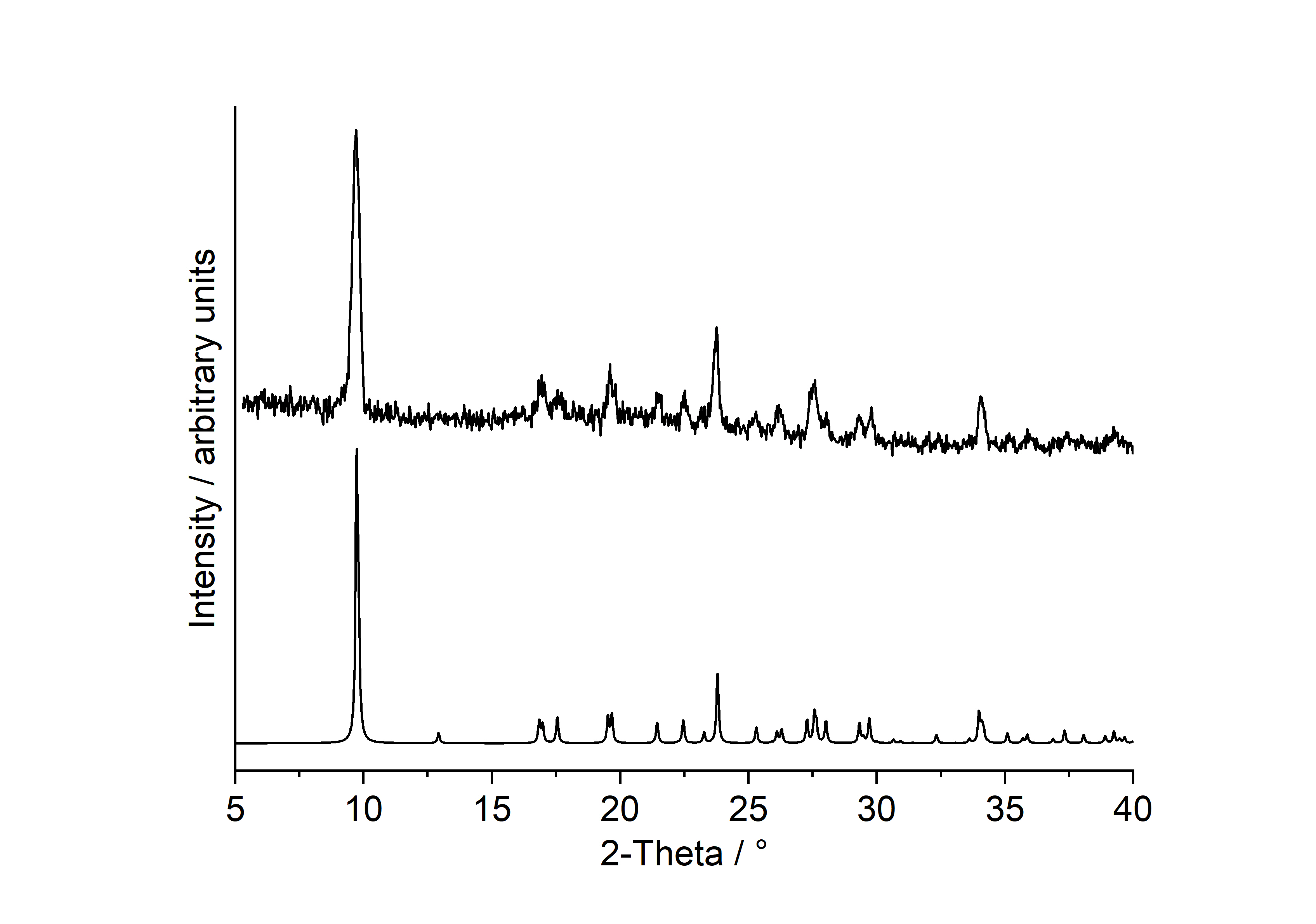

Supplement: Supplementary file 5 [file e-79-00167-sup5.jpg]

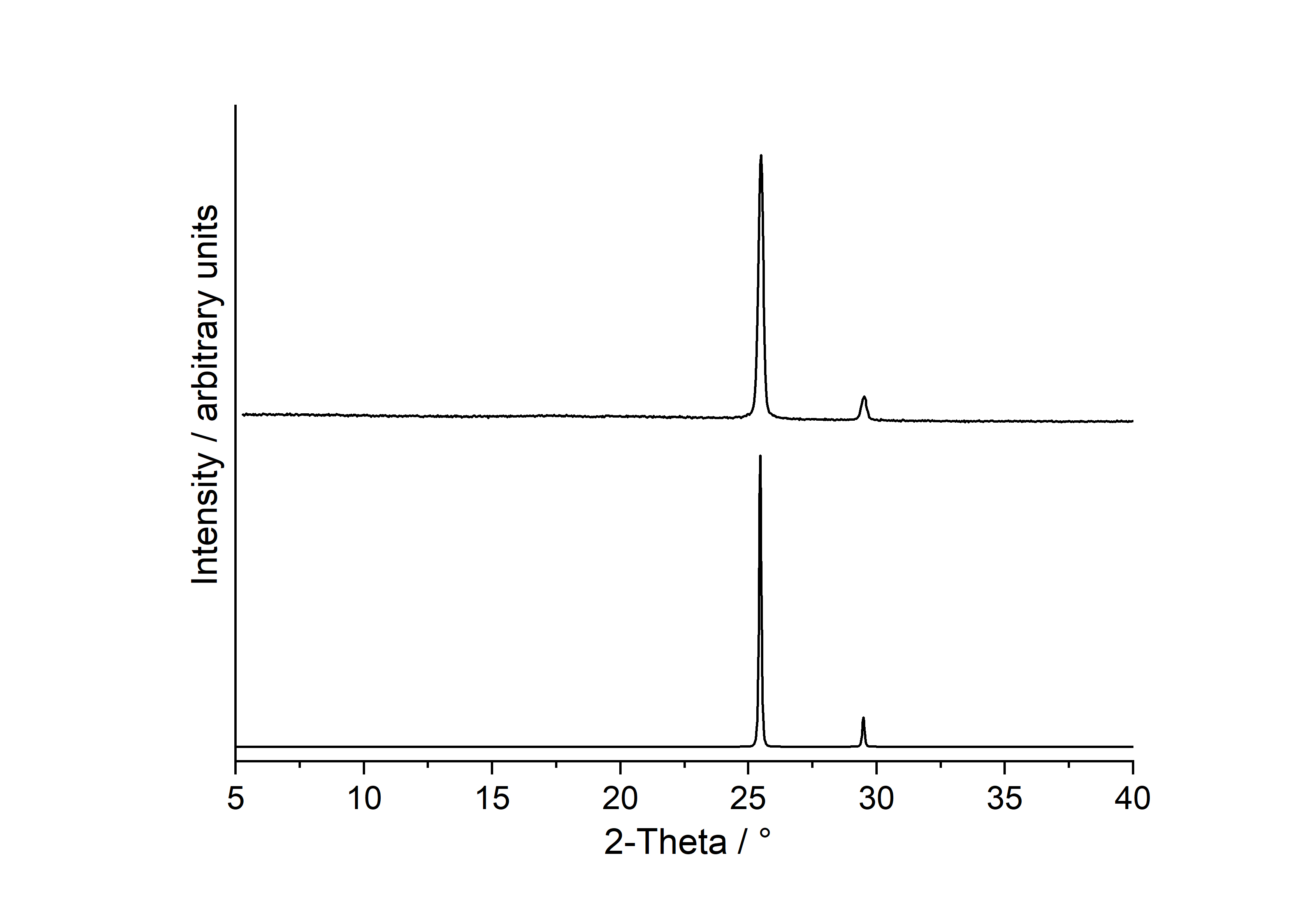

Supplement: Supplementary file 6 [file e-79-00167-sup6.jpg]
